# Vaginal Microbiota of Healthy Pregnant Mexican Women is Constituted by Four Lactobacillus Species and Several Vaginosis-Associated Bacteria

**DOI:** 10.1155/2011/851485

**Published:** 2011-09-22

**Authors:** César Hernández-Rodríguez, Roberto Romero-González, Mario Albani-Campanario, Ricardo Figueroa-Damián, Noemí Meraz-Cruz, César Hernández-Guerrero

**Affiliations:** ^1^Departamento de Microbiología, Escuela Nacional de Ciencias Biológicas, Instituto Politécnico Nacional, Plan de Ayala s/n esq. Prol. Manuel Carpio, Colonia Santo Tomás, C.P. 11340 México, DF, Mexico; ^2^Departamento de Salud, Universidad Iberoamericana, Ciudad de México, Prol. Paseo de la Reforma 880, Colonia Lomas de Santa Fe, C.P. 01219 México, DF, Mexico; ^3^Dirección de Enseñanza, Instituto Nacional de Perinatología “Isidro Espinosa de los Reyes” Montes Urales 800, Colonia Lomas Virreyes, C.P. 11000 México, DF, Mexico; ^4^Departamento de Infectologia e Inmunología Perinatal, Instituto Nacional de Perinatología “Isidro Espinosa de los Reyes” Montes Urales 800, Colonia Lomas Virreyes, C.P. 11000 México, DF, Mexico; ^5^Laboratorio de la Dirección de Investigación, Instituto Nacional de Perinatología “Isidro Espinosa de los Reyes” Montes Urales 800, Colonia Lomas Virreyes, C.P. 11000 México, DF, Mexico

## Abstract

*Objective*. To identify the microbiota communities in the vaginal tracts of healthy Mexican women across the pregnancy. *Methods*. Vaginal swabs were obtained during the prenatal visit of women from all trimesters (*n* = 64) of healthy pregnant women of Mexico City. DNA was isolated from each sample, and PCR-DGGE and sequencing of 16S rRNA gene fragments were used to identify the bacterial communities. *Results*. 21 different microorganisms were identified in the vaginal samples. *Lactobacillus* genus was present in 98% of women studied. Four lactobacilli species were identified in vaginal samples. *L. acidophilus* was the predominant (78%) followed by *L. iners* (54%), *L. gasseri* (20%), and *L. delbrueckii* (6%). 17 different microorganisms related to bacterial vaginosis conditions were identified. *Ureaplasma urealyticum* was the predominant (21%) followed by BVAB1 (17%) and *Gemella bergeriae* (7.8%). *Conclusions*. *Lactobacillus* genus predominates in the vaginal samples of Mexican pregnant women associated with different microorganisms related to bacterial vaginosis conditions.

## 1. Introduction

The healthy human vaginal microbiota in pregnant women plays a pivotal role in reproductive health and disease. The normal biota may prevent colonization of the host by pathogens and the spread of microorganisms related to urogenital infections, including those responsible for bacterial vaginosis. A disturbed vaginal microbiota is primarily associated with preterm labor, preterm rupture of membranes, and an increased risk of maternal and fetal morbidity [1]. 

Several studies have shown that the natural vaginal microbiota of healthy women of reproductive age is dominated by *Lactobacillus* spp. 

These bacteria play a critical role in preventing the overgrowth of pathogens and pathogenic opportunistic bacteria. The antagonistic effect is mediated by molecules such as hydroxide peroxide, lactic acid, and bacteriocins, which display antibacterial activity against catalase-negative bacteria. H_2_O_2_ affects catalase-negative bacteria, but lactic acid and bacteriocins can affect catalase-negative as well as catalase-positive bacteria and *Candida albicans* specifically those responsible for bacterial vaginosis [2–4]. 

According to Nugent's classification, a score from 7 and 10 is considered bacterial vaginosis, a clinical condition dominated by the morphological identification of different Gram-negative and -positive bacteria, without evidence of *Lactobacillus *morphotypes. In contrast, a score from 0 and 3 is considered an undisturbed vaginal microflora dominated by the *Lactobacillus* genus, identified as the principal Gram-positive rods bacteria [5]. 

Cultivation-dependent methods have failed to properly characterize vaginal microbiological communities for the following reasons: the culture bias applies to the normal vaginal microbiota as well as to the disturbed vaginal microbiota, the naturally competitive conditions exhibited by microorganisms *in vitro *can spread into the culture media, the specific or selective media necessary for cultivation of a particular microorganism may be unavailable. 

Molecular methods have identified in the vagina of healthy, nonpregnant women the *Lactobacillus* genus living with a spectrum of bacteria including *Gardnerella*, *Enterococcus*, *Bifidobacterium*, *Staphylococcus*, *Corynebacterium*, *Streptococcus*, *Bacterioides*, *Mycoplasma*, *Escherichia*, *Peptostreptococcus*, *Ureaplasma*, *Veillonela,* and *Candida* species [6–10]. However, at present the spectrum of bacterial species resident in the vaginal tracts of healthy, pregnant women is not well defined.

In the last decade, molecular techniques based on the analysis of the 16S rRNA gene fragment have allowed the identification of phylogenetically diverse microorganisms living in a precise ecosystem. PCR-denaturing gradient gel electrophoresis (PCR-DGGE) is a rapid and reliable molecular technique that has been applied to characterize the bacterial communities present in different biological niches, including the human vagina, gut, gingival, and skin [11–14].

The aim of present work was to characterize the vaginal bacterial communities present in Mexican women with a Nugent's 0–3 classification by PCR-DGGE and sequencing of 16S rRNA gene fragments in a transversal study. 

## 2. Material and Methods

### 2.1. Patients and Biological Samples

Healthy, pregnant women without vaginal bleeding, clinical symptoms of vaginal infection, or evidence of *Candida* colonization were enrolled in the study during routine prenatal examinations at the National Institute of Perinatology, Mexico City. Gestational age was estimated from the last menstrual period and early gestational fetal ultrasonographic measurements. To be eligible, women had to be free of subjective complaints, vaginal bleeding and oral or local antimicrobial therapies within the four weeks prior to enrollment. 

During the prenatal care visit, a vaginal sample was taken from 140 women in different weeks of pregnancy in a transversal study. A sterile speculum was inserted into each patient, and a sample from the posterior fornix of the vagina was collected using a Dacron sterile hyssop. Smears were made on microscope slides from vaginal swabs collected from each subject. The slides were Gram-stained and scored by Nugent criteria [5]. A score of 0 to 10 was assigned, considering the relative proportions of large Gram-positive, small Gram-negative, Gram-variable, and curved Gram-variable rods. Only women with a score of 0 to 3 were interpreted as having normal microbiota and were included in the study. The protocol was revised and approved by the internal institutional ethical and academic committee. Signed informed consent was obtained from all participants.

### 2.2. DNA Extraction

Total DNA of the vaginal samples was extracted using DNAzol reagent (Invitrogen, Carlsbad, Calif, USA), following the specifications provide by the manufacturer. DNA quality was estimated by electrophoresis in 1% agarose gels in TBE buffer (89 mM Tris, pH 8.3; 89 mM boric acid; 2 mM EDTA) and staining with 0.5 *μ*g/mL ethidium bromide. DNA concentrations and A260/A280 were determined spectrophotometrically with a Lambda 1A spectrophotometer (Perkin Elmer, Waltham, Mass, USA). An A260/A280 ratio of 1.8–2.1 was considered acceptable.

### 2.3. PCR-DGGE and Taxonomic Analysis of Vaginal Strains Based on 16S rRNA Gene Fragments Sequences

The diversity of the bacterial communities in each vaginal sample was studied by PCR-DGGE analysis. The V3 variable region of each bacterial 16S rRNA gene fragment was amplified by Muyzer technique [15] using 50 ng of metagenomic DNA from vaginal smears and the primers MAR-1 (5′-*CGC CCG CCG CGC GGC GGG CGG GGC GGG GGC *ACG GGG CCT ACG GGA GGC AGC AG-3′) and MAR-2 (5′-ATT ACC GCG GCT GCT GG-3′). The PCR consisted of 2.5 *μ*L of 10x PCR buffer (10 mM Tris-HCl, 2.5 mM MgCl_2_ and 50 mM KCl), 40 pmol of each primer, 0.8 mM of each deoxyribonucleoside triphosphate, 0.5 *μ*L (5 U) of Taq DNA polymerase and 1.5 *μ*L (50 ng) of template DNA solution in a final volume of 25 *μ*L. PCR was carried out for 35 cycles in a thermal gradient cycler (Eppendorf Scientific Inc., Westbury, NY, USA) with a denaturation step of 92°C for 45 s, followed by an annealing step at 55°C for 30 s and an extension step at 72°C for 45 s. A final extension step at 72°C for 7 min was added for all reactions. The expected size of the amplified fragment was 240 bp. In our research group, this PCR-based procedure has been frequently validated using as target bacterial genomic DNA from proteobacteria to sulphate-reducing bacteria and other taxons [16, 17]. DGGE analysis was performed with a D-Code Universal Detection System (Bio-Rad Laboratories, Hercules, Calif, USA). The linear denaturant gradient was attained using a communicating vessel gradient with a 16-cm gel that was 1 mm wide. PCR amplification products (25 *μ*L) were loaded into each well of the gel. Gels were run at 60 V for 16 h and maintained at 60°C in 1x TAE buffer (40 mM Tris, 20 mM acetate, 1 mM EDTA). At the end of the experiment, DNA separated in the DGGE gels was stained with a 1 : 10,000 dilution of reactive Vistra Green (Amersham Biosciences, Piscataway, NJ, USA) diluted in 50 mL of 1x TE buffer, pH 7.5, for 30 min. All visible DGGE bands were excised from gels with a sterile scalpel and placed into single Eppendorf tubes. Gel pieces were washed once in 1x PCR buffer and incubated overnight in 20 *μ*L of the same buffer at 4°C. Five microliters of the buffer solution was used as a template for PCR reamplification. The eubacterial primers without GC clamps and the PCR amplification conditions mentioned above were used for reamplification of each excised band from the DGGE gels. Reamplified bands were purified using the DNA Clean and Concentrator Kit (Zymo Research, Orange, Calif, USA) and sequenced by dideoxy chain termination. All sequences obtained in this work were subjected to a BLAST version 2.2.3 search [18] to assess the taxonomic hierarchy of the sequences and to select the related 16S rDNA bacterial sequences. Multiple alignment analyses with CLUSTAL X [19] were performed using the acquired sequences in this work and the related sequences selected from the NCBI Taxonomy Homepage (TaxBrowser). The identities of the sequences were determined on the basis of the highest percentage (a minimum of 95%) of total nucleotide match in GenBank.

### 2.4. Statistical Analysis

Patient characteristics, time of vaginal swab collection of enrolled women in the study, and infant-birth characteristics were analyzed by the Kuskal-Wallis one-way ANOVA; *P* < 0.05 was accepted as a significant difference. Statistical analysis was carried out with Sigma Stat software (Systat Software Inc., San Jose, Calif, USA).

## 3. Results

### 3.1. Characteristics of Women Included in the Study

A total of 64 samples from pregnant women with normal vaginal flora according Nugent's score (0–3) were included in the study. Women showed a mean of: 27.5 ± 6.7 years of maternal age; 38.4 ± 1.98 weeks at vaginal delivery; 21.5 ± 9.6 weeks at vaginal swab collection. Obstetric history showed a median of: 2 (1–5; (min-max)) gravities; 0 (1–5) vaginal deliveries; 0 (1–3) abortions; 0 (1–3) caesareans. Infant birth characteristic showed a mean of: 3045 ± 500.16 g weight outcome. Sixteen samples were from first trimester (25%), twenty five were from second trimester (39%), and twenty three were from last trimester (36%).  Table 1 shows the information respect to maternal age, obstetric history or infant birth characteristics at delivery of women included in the study rated by trimesters of pregnancy.

### 3.2. Identification of Vaginal Microbiota

**Figure 1:**
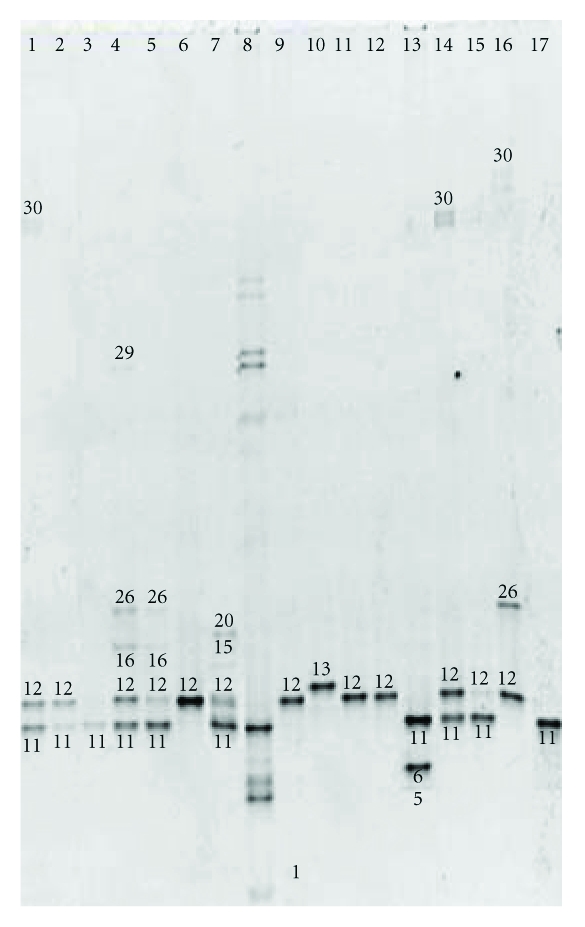
Denaturing gradient gel electrophoresis of vaginal samples from first trimester (*n* = 16). Lines 1–7 and 9–17 samples. Line 8 internal DNA lab standard. Number of DNA-band in the figure related to Table 2, where the percentage of identification in women studied and GenBank access number data identification are described.

**Figure 2:**
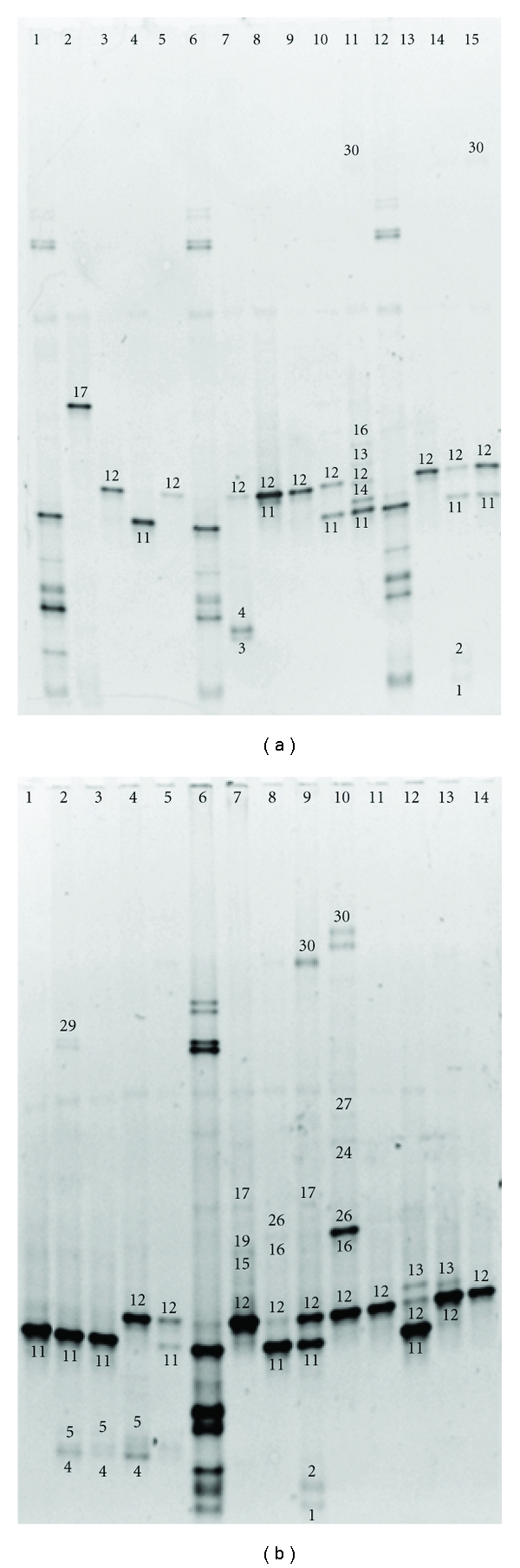
Denaturing gradient gel electrophoresis of vaginal samples from second trimester. Gel A (*n* = 12); lines 2–5, 7–11, and 13–15 samples. Lines 1,6,12 internal DNA lab-standard. Gel B (*n* = 13); lines 1–5 and 7–14 samples. Line 6 internal DNA lab-standard. Number of DNA-band in the figure related to Table 2, where the percentage of identification in women studied and GenBank access number data identification are described.

**Figure 3:**
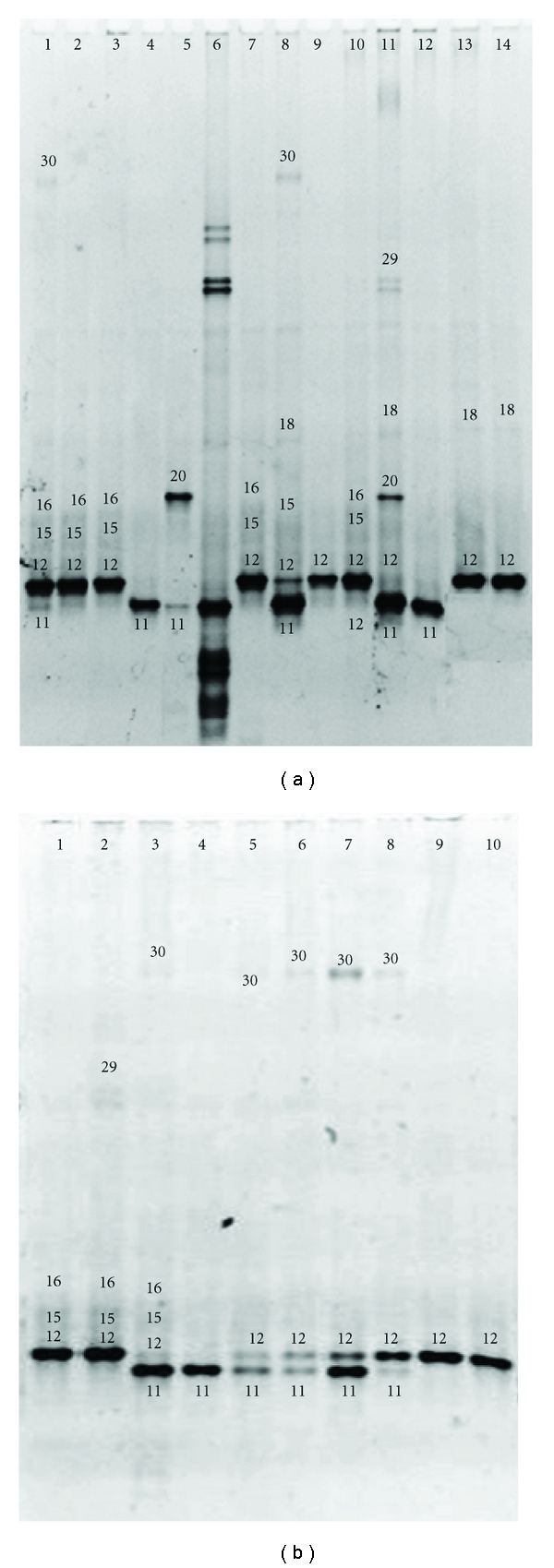
Denaturing gradient gel electrophoresis of vaginal samples from third trimester. Gel A (*n* = 13); lines 1–5 and 7–14 samples. Line 6 internal DNA lab standard. Gel B (*n* = 10); lines 1–10 samples. Number of DNA band in the figure related to Table 2, where the percentage of identification in women studied and GenBank access number data identification are described.

DGGE-DNA profiles of vaginal samples from 64 women included in the study are show in Figures 1, 2, and 3. Each DNA band in the figures has a number related to Table 2 where the diversity of microbiota identified in vaginal samples and GenBank access number data identification are described.

BLAST analysis of DNA sequences obtained from DGGE excised gel bands from 64 women correspond to 21 different bacterial species. *Lactobacillus* genus was detected in 63 of 64 women included in the study, only in one woman (1.5%) was not possible identified any species of *Lactobacillus* genus, solely *Peptostreptococcus *sp. was identified in that woman (Figure 2(a), lane 2). 

The *Lactobacillus* members were grouped into four species, with *L. acidophilus* being the most abundant (78.12%), followed by *L. iners* (54.68%), *L. gasseri* (20.31%), and *L. delbrueckii* (6.25%). *L. delbrueckii* was the most exiguous species in the vaginal tract, given that it was detected only in four samples (Table 2). 

**Figure 4:**
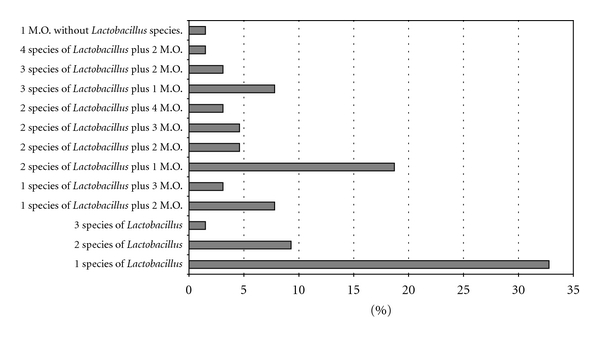
Profile of microorganisms identified in vaginal tract of women studied (*n* = 64). M.O. = microorganism (any microorganism different of *Lactobacilli* genus).

43% of women were colonized by one, two, or three different *Lactobacillus* species. 10.9% were colonized by one *Lactobacillus* species plus 1 or 2 different microorganism species. 31% of women were colonized by two *Lactobacillus* species plus 1, 2, 3, or 4 different microorganism species. 10.9% of women were colonized by three *Lactobacillus* species plus 1 and 2 different microorganism species. 1.5% of women (one woman) were colonized by 4 *Lactobacillus* species plus 1 different microorganism species (Figure 4). 

Taking into account the total number of 21 microorganism species identified (correspond to 163 bands amplified and sequenced from 64 women), 102 bands corresponded to the *Lactobacillus* genus (62.5%), 14 bands for uncultured *Ureaplasma urealyticum* (8.5%), 11 bands for BVAB1 (6.7%), and 5 bands for *Gemella bergireae* (3.0%). With respect to the remaining 31 bands they corresponded to ten bacteria which account for approximately 20% of the total microorganisms identified in the vaginal tract with individual values between 1% to 6%.

**Figure 5:**
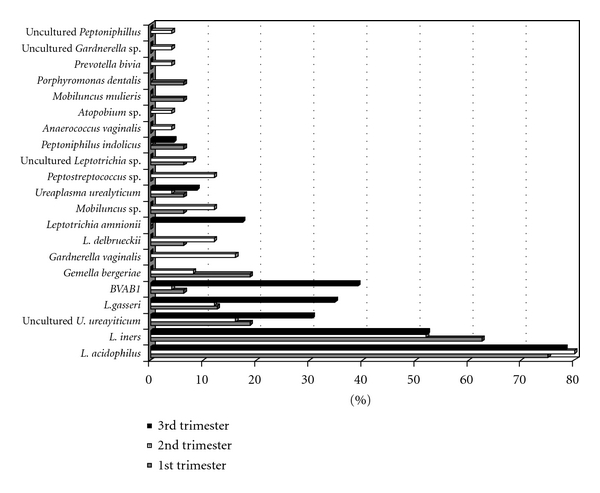
Distribution of 21 different microorganisms identified in vaginal tract of women studied (*n* = 64) by trimester of pregnancy.

Two microorganism (*Porphiromonas dentalis* and *Mobiluncus mulieris*), seven (*Atopobium* sp., *Gardnerella vaginalis*, *Prevotella bivia, Peptostreptococcus* sp., uncultured *Peptoniphilus* sp., *Anaerococcus vaginalis,* and uncultured *Leptotrichia* sp.), and one (*Leptotrichia amnionii)* were identified for first, second, and third trimesters, respectively (Figure 5). 

The major diversity of microorganism species was detected in vaginal samples from women enrolled in the second trimester, as samples from this stage contained sixteen bacteria from the total number of species identified. Also, twelve and eighth microorganisms were recognized molecularly in vaginal samples from women enrolled in the first and third trimesters, respectively (Figure 5).

## 4. Discussion

Human vaginal flora plays a profound role in reproductive health. Nevertheless, given the current limitations in our diagnostic abilities, it is naive to assume that we know all microorganisms present the vaginal tract in healthy and unhealthy pregnancies; the present paper aims to attend to this concern. 

*Lactobacilli* genus was present in vaginal samples from all pregnant women studied except for one woman of second trimester, who showed *Peptostreptococcus *sp., as the only identified microorganism. On the other hand, a very similar distribution with respect to the *Lactobacillus* species was observed in the remaining women. *L. acidophilus* was the most abundant microorganism (78%), followed by *L. iners* (54%), *L. gasseri* (20%), and* L. delbrueckii* (6%). 

Very few studies have been published with respect to the vaginal microbiota in healthy and unhealthy pregnancies. In 2007, Kiss et al. studied 126 healthy, pregnant Swedish women (Nugent score 0–3) between 11 to 14 gestational weeks and applied a species-specific PCR technique on vaginal samples. The author identified the presence of eight different lactobacilli species, with *L. gasseri* (26.4%), *L. crispatus* (23.6%), *L. jensenii* (19.4%), and *L. rhamnosus* (9.7%) being the most abundantly observed species [20]. However, the author did not detect *L. acidophilus*, the lactobacilli species most frequently detected in our study. Likewise, in 2007, Tamrakar et al. [21] studied 98 healthy, pregnant Japanese women between 5 to 36 gestational weeks (mean of 23 weeks) and applied a species-specific PCR technique for fourteen *Lactobacilli* species on vaginal samples. Four lactobacilli, *L. crispatus* (61.2%), *L. jensenii* (29.6%), *L. gasseri* (33.7%), and *L. iners* (39.8%), showed the highest prevalence in the vaginal samples. *L. delbrueckii* was not detected, and *L. acidophilus* was not included as a target of the study. 

Our results agree with the two authors mentioned above in that the four discussed *Lactobacilli* species were the most abundant microorganisms observed across all trimesters of pregnancy. However, we cannot recognize *L. crispatus* and *L. jensenii* which were identified in the previous mentioned manuscripts and are the most predominant species worldwide reported. 

On this respect, using the same conditions and PCR procedures described in Material and Methods section, we confirmed that the primers can amplify these species from a cultured strain (data not shown) and produce DGGE fragments with the expected molecular size, which can be distinguished from those of other species after sequence the DGGE fragments and apply the bioinformatic analyses. This evidence demonstrates that the PCR-DGGE strategy is proper to recognize *Lactobacillus* spp. DNA target.

On the other hand, the absence of *L. crispatus* and *L. jensenii* in Mexican population samples is a surprising data that must be confirmed. However, an independent study reveals that both species were not frequently isolated from Mexican population (Castro-Escarpulli G., personal communication). Also, culture independent studies have not detected *L. jensenii *[22], and relevant differences in the composition of vaginal microbial communities, particularly *Lactobacillus* spp., have been found in healthy Caucasian and black women [23]. 

Although few data have been reported in this area, vaginal *Lactobacillus* spp. distributions can vary across specific groups, perhaps as a consequence of ethnic conditions, food intake, behavior, habits, and customs [24–27]; evidently, more information must be accumulated.

Molecular studies performed with nonpregnant healthy women have shown a limited* Lactobacillus *diversity in the vagina that is restricted to three to seven species, with the specific distribution of lactobacilli species being dependent on the group of women studied [28, 29]. 

On the other hand, a different distribution of microorganisms related to bacterial vaginosis conditions [30–32] were characterized in the vaginal tract of women studied. Several manuscripts, where molecular techniques were applied to evaluate disturbed vaginal tract conditions, have shown a wide distribution of vaginosis-associated bacteria (VAB), with a clear decrease in the number and/or abundance of protective lactobacilli species [33–35]. Our data showed a wide distribution of VAB in vaginal samples of women studied; however, clinical data and morphological vaginal characterization of smears by Nugent's criteria were compatible with healthy vaginal tract. Despite any experiment was done to evaluated the protective effect of lactobacilli group in the women studied, we think that *Lactobacillus* species confer protection against the overgrowth of potentially pathogenic bacteria by means of the release of metabolic products such as H_2_O_2_, lactic acid, and bacteriocins as have been described previously [2–4], which maintain the status of normal vaginal microbiota inhibiting the colonization and spreading of local or transit pathogens. 

In our results, the pattern of distribution of lactobacilli species was very constant among studied women, since 33% of women showed *L. acidophilus*, followed by the pair of *L. acidophilus* plus *L. iners* (8%) and the triad of *L. acidophilus* plus *L. gasseri* (2%) as the microorganisms only identified in vaginal samples. This pattern of distribution of *Lactobacillus* species was the same even though vaginosis-associated bacteria were detected in vaginal samples, since women with *L. acidophilus* plus 2 and 3 VAB account 9%, the *L. acidophilus*/*L. iners* plus 2–4 VAB account 29% and the triad *L. acidophilus/L. iners/L. gasseri* plus 1 and 2 VAB account 10%. This data support the idea of that a specific group of *Lactobacillus *species in vaginal tract of women prevents the spread of microorganisms potentially capable to cause urogenital infections, including those responsible for bacterial vaginosis. 

In this respect, a manuscript published by Verstraelen et al. [36] demonstrated in a prospective study of pregnant women that the presence of specific lactobacilli species in the vaginal tract of healthy women is a pivotal or protective factor for the conversion to abnormal microbiota evaluated by Gram stained smears. The presence of *L. crispatus* alone in the vaginal tract of healthy women or accompanied with other lactobacilli species as *L. jensenii, L. gasseri* and *L. iners* confers a protector effect (RR 0.2; 95% CI 0.05–0.89) to development an abnormal vaginal microbiota, against the presence of *L. gasseri/iners* who account an increased risk (RR 10.41; 95% CI 1.39–78.12) for the conversion to abnormal vaginal microbiota. 

The data present herein showed a characteristic pattern of *Lactobacillus* species in healthy women even when different vaginosis-associated bacteria were detected in vaginal samples. Although the study design of the present paper and the Verstraelen is different, the comparison of *Lactobacillus* species found in our paper and the *Lactobacillus* species reported by the author in the vaginal tract of healthy women (Grade I) of first trimester, showed a similar distribution respect to the number of *Lactobacillus* species detected, since the author reported 67% of the women colonized by one species of lactobacilli, 24.7% by two species and 6.5% by three and four species. 

The paper present herein adds information respect to the *Lactobacillus* genus that resides in the vaginal tract of Hispanic women. In this area, improved knowledge of normal microbiological species present in the vaginal tracts of healthy, pregnant women in a particular population could aid in the development of specific probiotic and prebiotic therapies as well as prophylactic alternatives to help patients avoid vaginosis-associated deleterious fetomaternal outcomes.

## 5. Conclusions

Twenty one different bacteria species were detected in vaginal samples from healthy women. The *Lactobacillus* genus was detected in 63 of 64 women included in the study. The lactobacilli members were grouped into four species, with *L. acidophilus* being the most abundant (78.12%) followed by *L. iners* (54.68%), *L. gasseri* (20.31%), and *L. delbrueckii* (6.25%). Seventeen different microorganisms related to disturbed or bacterial vaginosis conditions were identified in the vaginal tract of pregnant women, with dissimilar distributions among studied women. Uncultured *U. realyticum* was the most abundant microorganism (21%) followed by BVAB1 (17%) and *Gemella bergirae* (7.8%). Fourteen remain microorganisms showed prevalence between 1 to 6%.

## Figures and Tables

**Table 1:** Pregnant women and infant-birth characteristics in each trimester of study.

	First trimester (*n* = 16)	Second trimester (*n* = 25)	Third trimester (*n* = 23)
*Maternal Age* (y)*	28.8 ± 6.0	27.4 ± 8.2	26.7 ± 5.3
	(28; 15–37)	(28; 13–43)	(27; 17–37)

Obstetric history**			
Gravity	2 (1–5)	2 (1–5)	2 (1–5)
Vaginal delivery	1 (0–5)	0 (0–3)	0 (0-1)

Infant/birth characteristics*			
Gestational age at delivery (wk)	37.9 ± 1.4	38.2 ± 2.5	38.9 ± 1.5
	(38; 37–40)	(38; 37–41)	(39; 37–42)
Infant weigh outcome at delivery (g)	3033 ± 393	2974 ± 656	3132 ± 355
	(2955; 2520–3960)	(3110; 2580–3900)	(3130; 2520–3800)

*Weeks at vaginal* ^∗,+^ *swab collected*	10.1 ± 1.3	18.5 ± 2.8	32.8 ± 4.2
	(10; 7–12)	(18; 13–24)	(33; 27–41)

*Data are given in mean ± SD with median and ranges in parenthesis. **Data are given in median with ranges in parenthesis. **^+^** *P* < 0.05; data compared with Kuskal-Wallis one-way ANOVA.

**Table 2:** Microorganisms identified in the vaginal tract of pregnant healthy women.

Number of DNA band shows in the figures	Name of microorganism	GenBank access number	Times identified in women (*n* = 64)	Percentage of women with species identified (%)
12	*Lactobacillus acidophilus*	NC_006814	50	78
11	*Lactobacillus iners*	AY283265	35	55
30	Uncultured* Ureaplasma urealyticum *	EU644473	14	22
16	*Lactobacillus gasseri*	NC_008530	13	20
15	BVAB1	AB034121	11	17
26	*Gemella bergeriae*	Y13365.1	5	8
4	*Gardnerella vaginalis*	M58744	4	6
13	*Lactobacillus delbrueckii*	NC_008529	4	6
18	*Leptotrichia amnionii*	AY078425	4	6
5	*Mobiluncus *sp.	EF428974.1	4	6
29	*Ureaplasma Urealyticum*	AF073455	4	6
17	*Peptostreptococcus* sp.	AY207059	3	5
1	Uncultured *Gardnerella *sp.	AY738665.1	3	5
20	*Peptoniphilus indolicus*	D14147	2	3
24	*Anaerococcus vaginalis*	AF542229	1	2
2	*Atopobium *sp.	AY738658.1	1	2
6	*Mobiluncus mulieris*	AJ427625	1	2
3	*Porphiromonas dentalis*	X81876.1	1	2
14	*Prevotella bivia*	L16475	1	2
27	Uncultured *Leptotrichia *sp.	AY724742.1	1	2
19	*Uncultured Peptoniphilus *sp.	AY738692.1	1	2

Percentages in the column of “% of women with species identified” were rounded off to whole percents.
